# Personality and spatial familiarity interact to shape affective responses in urban parks: a VR study

**DOI:** 10.3389/fpsyg.2026.1700552

**Published:** 2026-04-14

**Authors:** Jie Liu, Hanyang Hu, Mengyin Jiang, Yunpeng Jia

**Affiliations:** 1 School of Digital Media and Design Arts, Beijing University of Posts and Telecommunications, Beijing, China; 2 School of Architecture, Tsinghua University, Beijing, China; 3 Deep Space Exploration Laboratory/National Key Laboratory for Deep Space Exploration, Hefei, China; 4 Department of Social and Behavioural Sciences, City University of Hong Kong, Kowloon, Hong Kong SAR, China

**Keywords:** affective responses, spatial familiarity, heart rate variability, personality traits, virtual reality

## Abstract

With rapid urbanization, mental health issues among urban residents have become increasingly prominent, and urban parks as important green spaces hold potential value for promoting public mental health. However, prior research has largely focused on environmental physical characteristics, overlooking how the interaction between spatial familiarity and individual personality traits shapes affective experiences. This study employed virtual reality (VR) technology, wherein 72 participants experienced two urban parks differing in familiarity, assessing affective responses via the Positive and Negative Affect Schedule (PANAS) and heart rate variability (HRV). The findings revealed that personality traits significantly predicted affective responses. Extraversion was associated with higher positive affect (PA), whereas neuroticism was associated with higher negative affect (NA). Spatial familiarity also showed a main effect, with lower familiarity associated with increased NA. Crucially, significant interaction effects indicating that spatial familiarity moderated the influence of personality traits on NA. In less familiar parks, individuals with higher neuroticism exhibited more NA, while greater conscientiousness and openness alleviated these negative responses (*p* < 0.05). Multiple linear regression analysis confirmed neuroticism and openness as strong predictors of negative affect in low-familiarity parks (*R*-squared = 0.260, *p* < 0.01). HRV measurements demonstrated that urban parks consistently reduced affective arousal levels in participants, regardless of familiarity. These findings highlight the multilevel interaction between personality and environmental experience in affective restoration, advancing understanding of psychological mechanisms underlying urban park exposure and emphasizing the role of human factors in shaping emotional responses.

## 1 Introduction

Urban parks, which are essential green spaces for relaxation, social interaction, and physical activity, are indispensable for urban “residents” physical and mental wellbeing. With the acceleration of urbanization and the increasing pressures of daily life and work, the demand for accessible natural environments, particularly urban parks, has intensified (Hedblom et al., 2019; Wang and Chang, 2023). A substantial body of research has demonstrated that exposure to urban green spaces can effectively alleviate stress and elicit positive affective responses, thereby enhancing overall happiness and psychological health (Harris et al., 2025; Zhu et al., 2023). Consequently, numerous studies have examined how specific environmental characteristics, such as vegetation density (Wang and Li, 2024), spatial openness (Wang et al., 2025), water features (Liu and Liu, 2025), and sensory qualities (Chen and Ma, 2025), shape restorative experiences in parks. However, accumulating empirical evidence suggests that the same park environment does not produce uniform affective outcomes across individuals (Sella et al., 2023; Mizumoto et al., 2025). Even under identical environmental conditions. Visitors often report markedly different emotional experiences and stress recovery levels (Yang et al., 2024; Jin et al., 2022). These pronounced inter-individual differences indicate that environmental attributes alone are insufficient to fully explain affective responses to urban parks, highlighting the need to consider human-related factors in restorative environment research.

Among various human-related factors [gender (Sang et al., 2016), age (Pavic et al., 2024), educational background (Liu Q. et al., 2022)], personality traits have been increasingly identified as a key determinant influencing how individuals perceive, evaluate, and emotionally respond to natural environments (Sella et al., 2023). Personality psychology suggests that traits such as neuroticism, extraversion, agreeableness, openness, and conscientiousness (de Raad and Mlaèiæ, 2015) shape emotional sensitivity, environmental appraisal, and stress regulation processes. For example, extraversion has been consistently linked to higher positive affect (PA), while neuroticism is strongly associated with higher negative affect (NA) (DeNeve and Cooper, 1998; Mora et al., 2019). Nevertheless, empirical findings regarding the role of personality traits in green space-related emotional restoration remain inconsistent. While some studies associate lower neuroticism with greater psychological stress reduction (Wang et al., 2024), others report stronger restorative effects among individuals with higher neuroticism (Johnsen, 2013) or non-significant effects (Kley et al., 2025; Sella et al., 2023). Similarly, the influence of extraversion (Senese et al., 2019; Saw et al., 2015) and conscientiousness (Seiken, 2022; Johnsen, 2013) on affective recovery varies across studies and contexts. These inconsistencies suggest that personality traits alone may not directly determine restorative outcomes, pointing instead to the presence of moderating or mediating mechanisms that remain insufficiently explored.

One potential mechanism that may help explain these divergent findings is spatial familiarity. From a cognitive-neuropsychological perspective, brain regions involved in meaning-making, memory, and visual appreciation interact closely with limbic structures responsible for affective processing (Chatterjee, 2013). Familiarity, largely grounded in recognition memory, has therefore been widely used to investigate individuals’ experiential responses to different environments. Prior research indicates that familiarity not only fosters environmental preference (Terzano and Gross, 2020; Lau et al., 2024) but also motivates repeated visitation and sustained proximity to specific places (Scannell and Gifford, 2010). In the context of urban parks, familiar environments have been shown to promote a stronger sense of place attachment (Bazrafshan et al., 2023; Lebrusán and Gómez, 2022), which is closely associated with feelings of comfort, safety, and relaxation. Moreover, cultural background (Zhao Z. et al., 2025) and childhood experience (Koivisto and Grassini, 2022) can shape environmental evaluations, likely because prior exposure and environmental expectations influence perceived familiarity and preference. These observations suggest that familiarity may moderate the relationship between personality traits and affective responses in urban parks.

Importantly, evidence from prior research suggests that familiarity is unlikely to exert uniform effects across individuals (Terzano and Gross, 2020). Its emotional benefits may interact with personality-related affective tendencies (Lionetti and Pluess, 2024). For instance, individuals high in neuroticism, who are more sensitive to uncertainty and potential threat cues, may derive greater emotional security and reduced negative affect in familiar environments, whereas unfamiliar settings may amplify emotional discomfort. Such personality-familiarity interactions may partially account for the inconsistent findings regarding personality effects reported in prior green space research.

Building on these insights, the present study aims to investigate whether spatial familiarity moderates the relationship between personality traits and affective responses to urban parks. By integrating personality psychology with environmental familiarity theory, this study seeks to clarify the underlying mechanism through which individual differences shape emotional experiences in restorative environments.

## 2 Related works

### 2.1 Affective responses and emotional restoration in urban parks

Urban parks are widely regarded as essential components of urban green infrastructure that contribute to stress reduction, fatigue recovery, and emotional wellbeing. Early experimental studies demonstrated that exposure to natural scenes is associated with lower negative affect and higher positive affect compared with exposure to built environments lacking natural elements (Ulrich, 1979). Subsequent empirical research has consistently confirmed the restorative effects of urban parks and natural environments across diverse populations and settings, showing reductions in psychological stress, emotional fatigue, and attentional depletion (Tyrväinen et al., 2014; Wilkie and Clouston, 2015; Hazer et al., 2018; Jo et al., 2019; Yao et al., 2021). These effects are commonly interpreted through established theoretical frameworks, including the Biophilia Hypothesis, which posits an innate human affinity for natural environments (Fromm, 1964; Wilson, 1986), Attention Restoration Theory (ART), which emphasizes the recovery of directed attention through soft fascination (Kaplan and Kaplan, 1989; Kaplan, 1995) and Stress Recovery Theory (SRT), which highlights the rapid affective and physiological benefits of nature exposure (Ulrich et al., 1991).

Beyond *in situ* park experiences, an expanding body of research has demonstrated that mediated representations of natural environments can also elicit substantial affective and restorative benefits.

Viewing images of natural environments has been shown to reduce work-related stress and mental fatigue (van den Berg et al., 2015), while exposure to videos depicting natural settings has been associated with enhanced positive affect and attentional recovery compared with artificial environments (van den Berg et al., 2003). More recently, immersive virtual reality technologies have been increasingly adopted to simulate urban parks and natural environments under controlled conditions, enabling researchers to manipulate environmental stimuli while eliciting affective and physiological responses comparable to those observed in real-world exposure (Chan S. H. M. et al., 2023; Lyu et al., 2022; Mihara et al., 2023; Soltanzadeh et al., 2024; Xiaoxue and Huang, 2024). This methodological shift has facilitated more precise quantification of emotional responses and stress recovery processes, supporting the validity of VR-based approaches in restorative environment research.

Within this growing literature, quantitative analyses have predominantly focused on how variations in environmental stimuli shape affective outcomes. At the spatial and morphological level, studies have examined factors such as park size (Kong et al., 2022; Zhao Z. et al., 2025), spatial openness (Franěk, 2023), enclosure (Liu et al., 2025; Tabrizian et al., 2018), path configuration (Gao and Fang, 2025; Thwaites et al., 2005), accessibility (Zhao Z. et al., 2025), connectivity (Gao and Fang, 2025), and walkability (Nghiem et al., 2021; Petrunoff et al., 2021), demonstrating their associations with perceived comfort, relaxation, and emotional restoration. At the landscape composition level, research has focused on vegetation coverage (Liu et al., 2023), plant diversity (Wei et al., 2022), canopy density (Zeng et al., 2021), seasonal variation (Li et al., 2021; Da et al., 2024), color composition (Kaufman and Lohr, 2002), and the presence of water features (Gao and Fang, 2025; Kong et al., 2022), all of which have been linked to changes in positive affect, stress reduction, and perceived restorativeness. Functional and activity-related attributes, including opportunities for physical exercise (Han, 2017), outdoor gardening (van den Berg and Custers, 2011), social interaction (Wang et al., 2023), and leisure activities (Yang et al., 2025), as well as the availability of facilities and amenities (Chu et al., 2021), have also been shown to influence emotional experiences by shaping how individuals engage with park spaces. In addition, sensory qualities such as naturalness (Felappi et al., 2024), animal biodiversity (Nghiem et al., 2021), soundscapes (Zeng et al., 2021), olfactory cues (Taufer et al., 2025), and thermal comfort (Xin et al., 2024), as well as contextual and management-related factors including cleanliness (Veitch et al., 2025), maintenance quality (Chen et al., 2024), safety perception (Felappi et al., 2024), lighting conditions (Masullo et al., 2022), crowding (Zhan and Zheng, 2026), and ambient noise (Jin et al., 2024), have been incorporated into quantitative models to explain variations in affective responses.

Although environmental characteristics are important determinants of emotional restoration, previous studies indicate that affective responses can vary substantially across individuals exposed to the same park settings (Sella et al., 2023; Mizumoto et al., 2025). Visitors may experience markedly different levels of relaxation, positive affect, or stress reduction, suggesting that environmental attributes alone do not fully account for emotional outcomes. While demographic variables such as age (Wei et al., 2022), gender (Weng et al., 2025; Wei et al., 2022; Wang and Fei, 2025), and education (Liu Q. et al., 2022) have been examined in some studies, these factors primarily describe population characteristics rather than underlying psychological mechanisms. These observations highlight the value of incorporating individual psychological factors into research on emotional restoration. Stable dispositions, such as personality traits, and other individual differences, including connectedness to nature or prior experience with natural environments, may help explain why people respond differently to the same environmental stimuli.

### 2.2 Affective responses in urban parks across personality traits

Personality traits play a critical role in shaping individuals’ emotional sensitivity, appraisal, and stress regulation, thereby influencing affective responses to natural environments. Among various personality frameworks, the Big Five dimensions (neuroticism, extraversion, openness, agreeableness, and conscientiousness) play a crucial role in shaping emotional sensitivity, appraisal, and stress regulation (de Raad and Mlaèiæ, 2015). Neuroticism highlights a tendency toward emotional instability and negative emotions (Yang et al., 2020). Conscientiousness is characterized by robust self-discipline, trustworthiness, and a practical approach to planning and organization (Yang et al., 2020). Agreeableness assesses the extent of an individual’s kindness, cooperation, and trust in others. Openness to experience signifies a person’s innovative thinking, vivid imagination, and eagerness to explore new endeavors (Szcześniak et al., 2020). Extraversion is associated with the degree of sociability, confidence, and vitality that a person demonstrates in social settings (Chmielewski and Morgan, 2013; Wilt and Revelle, 2019). Consistent with broader research on personality and daily affective experience has shown that different personality traits can lead to varying affective experiences in daily life (Letzring and Adamcik, 2015). Specifically, extraversion, conscientiousness, and openness are positively related to PA, while neuroticism is positively associated with NA. Conversely, agreeableness is negatively associated with NA (Larsen and Ketelaar, 1989). These disposition-affected associations suggest that personality may shape how individuals engage with and benefit from restorative environments.

Empirical studies examining personality in the context of green space exposure provide further, albeit mixed, evidence. For example, extraversion has been identified as a significant predictor of perceived restorativeness following nature exposure, alongside connectedness to nature (Sella et al., 2023), while some immersive virtual reality research reported weak or even negative correlations between extraversion and specific restorative dimensions (Senese et al., 2019). High neuroticism, low extraversion, and low conscientiousness are considered vulnerability factors that may predispose individuals to anxiety and panic (Wardenaar et al., 2014), suggesting that these traits may shape individual differences in recovery from stress. Lower neuroticism has been linked to greater psychological benefits from green space exposure in some studies (Ambrey and Cartlidge, 2017), yet other research suggests individuals with higher neuroticism or internalising tendencies may experience greater restorative gains from quality green spaces, particularly among adolescents and females (Feng et al., 2022). In laboratory and field studies, conscientiousness and Agreeableness has occasionally been associated with perceived restorativeness (Sirois and Hirsch, 2015), but effects are not consistently robust across contexts (Sella et al., 2023). These mixed findings indicate that the influence of personality traits on affective responses in green environments may depend on sample characteristics, context, and measurement approaches.

Furthermore, personality dimensions have been shown to influence subjective evaluation of environmental features such as soundscapes, eventfulness, and perceived pleasantness (Lindborg and Friberg, 2016). For example, higher emotional stability is associated with a broader perception of eventfulness in natural soundscapes, while higher neuroticism corresponds to a narrower perception (Lindborg and Friberg, 2016). In contrast, higher openness relates to a narrower perception of eventfulness, and lower openness to a broader perception (Lindborg and Friberg, 2016). Extraversion increases tolerance to sound, whereas high noise sensitivity reduces it (Chan T. C. et al., 2023). Moreover, trait-based factors shaped by prior experience often have a stronger impact on perceived restorative effects of urban soundscapes than innate temperament (Jeon et al., 2021). Exposure to natural acoustic environments, such as birdsong, rustling leaves, and flowing water, enhances affective restoration, particularly when individual personality predispositions align with the environmental stimuli (Jeon et al., 2021). These findings indicate that auditory features interact with personality to shape perceived restorative potential and emotional responses in urban parks.

Building on these insights, it becomes evident that personality traits are relevant to emotional responses in natural settings, yet they alone cannot fully explain the variability in affective responses to urban parks. Individual differences in prior experiences, environmental expectations, and psychological dispositions may influence how restorative potential is perceived and experienced. These observations suggest that additional human-related factors could interact with personality traits to shape emotional outcomes in natural settings, pointing to the need for further research into the mechanisms that underlie inter-individual differences in green space benefits.

### 2.3 Interactive effects of spatial familiarity and personality traits on affective responses

Spatial familiarity refers to an individual’s comprehensive level of familiarity with a specific geographic place, shaped by the integration of their cognitive understanding, practical interactive experience, and affective perception of the place (Kitchin, 1994). In environmental psychology, familiarity is often examined through related constructs such as place attachment, which reflects enduring cognitive-emotional bonds between individuals and places (Lewicka, 2011; Scannell and Gifford, 2010). Within the Person-Place-Process (PPP) framework, familiarity can be understood as an integrative mechanism linking personal characteristics, environmental attributes, and affective-cognitive processes (Lewicka, 2011).

Existing research suggests that spatial familiarity may influence emotional experiences through several pathways. Familiar environments tend to reduce cognitive load and uncertainty, enabling attentional resources to support emotional regulation and recovery (Harms et al., 2021; Ratcliffe and Korpela, 2016; Liu et al., 2020; Reber et al., 2004). Greater predictability and perceived environmental mastery are associated with increased feelings of safety and control, which relate to lower stress responses (Meyer et al., 2021; Nicolas et al., 2022). Familiar settings are also more likely to evoke positive autobiographical memories and emotional associations, thereby enhancing relaxation and psychological comfort (Ratcliffe and Korpela, 2016; Bazrafshan et al., 2023). These mechanisms may be especially relevant in natural environments that already support cognitive and affective restoration.

Spatial familiarity is conceptually distinct from simple environmental preference. While preference reflects immediate liking, familiarity involves accumulated experience and meaning-making processes that shape emotional responses over time (Terzano and Gross, 2020; Nezlek, 2007). Higher familiarity is often associated with stronger feelings of belonging and emotional connection (Su et al., 2025; Berg, 2020), which may further enhance perceived restorative quality (Liu et al., 2020) and condition how personality traits relate to affective restoration. Individuals high in neuroticism, who are more sensitive to uncertainty and potential threat, may experience greater emotional benefits in familiar natural environments, whereas unfamiliar settings may attenuate restorative effects. In contrast, highly extraverted individuals may respond more positively to novelty and stimulation in less familiar environments. Evidence from research on spatial anxiety and navigation supports the notion that familiarity moderates emotional responses by influencing cognitive demand and affective states (Nori et al., 2023; Kim et al., 2021).

Despite its relevance, spatial familiarity has seldom been examined as a moderating factor between personality traits and affective restoration in natural settings. Most studies focus on direct associations between place attachment, preference, or exposure frequency and restorative outcomes, with limited integration of personality dimensions. Consequently, the extent to which spatial familiarity can account for inconsistent personality effects in green space research remains unclear. Examining their interaction may therefore rarely been examined as a moderating factor in the relationship to a more refined understanding of individual differences in emotional responses to urban parks.

### 2.4 Research question

Building on previous research, the present study examines whether spatial familiarity moderates the relationship between personality traits (the Big Five) and affective responses (positive and negative affect) in urban parks, and further explores how personality and familiarity jointly influence emotional restoration. To address this overarching question, we consider three sub-questions:

*Q1*: Do different personality traits (the Big Five) significantly relate to positive and negative affect in urban parks?

*Q2*: Does spatial familiarity independently influence affective responses in urban parks?

*Q3*: Does spatial familiarity moderate the relationship between personality traits and emotional responses, potentially altering the direction or strength of these effects?

Based on these questions, we hypothesize:

*H1*: Extraversion is positively associated with positive affect, while neuroticism is positively associated with negative affect in urban parks.

*H2*: Higher spatial familiarity is associated with increased positive affect and decreased negative affect in urban parks.

*H3*: Individuals with different personality traits will show distinct affective response patterns in urban parks of varying familiarity. Specifically, those with certain personality traits, such as high neuroticism, low extraversion, and low conscientiousness, may be more susceptible to negative emotions in low-familiarity environments.

## 3 Methods

### 3.1 Study sites and visual stimuli

**FIGURE 1:**
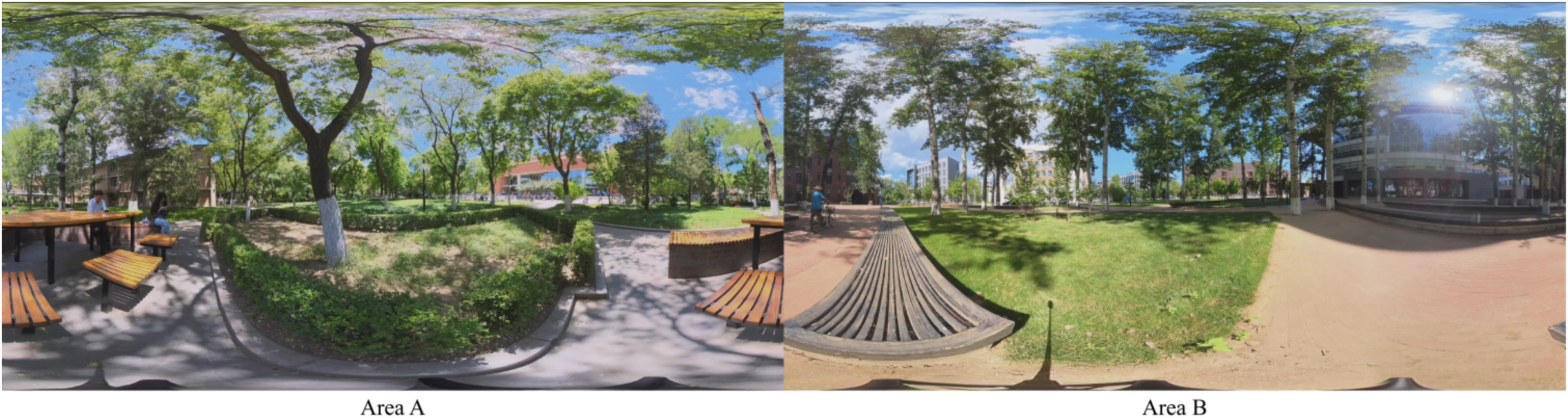
Characteristic panoramic views of Area A **(left)** and Area B **(bottom)** used in the experiment.

The study selected two representative green spaces, akin to urban parks, located within University A (Area A) and University B (Area B). These green spaces, integral to urban greening efforts, serve as urban parks for the students, faculty, and staff who reside, study, and work there, providing similar social and ecological functions. Both parks offer a typical urban park environment, featuring a variety of trees and shrubs, expansive lawns, open skies, resting benches, walking paths, and iconic campus buildings. To ensure consistent experimental conditions, we kept the green visual index for both scenes within the 40–55% range, thereby maintaining equivalent visual exposure to greenery (Figure 1).

As numerous studies and meta-analyses have demonstrated that high-fidelity panoramic VR is a reliable surrogate for field visits in environmental psychology research, eliciting physiological and affective responses comparable to those experienced in real-world settings (Higuera-Trujillo et al., 2017; Newman et al., 2022; Zhao Y. et al., 2025), we therefore adopted panoramic VR as the experimental medium. In our specific experimental design, since the two sites in the experiment are urban parks in the same city, the auditory soundscapes, such as bird songs and trees sound, are highly similar. Auditory stimuli generally exert a weaker influence on restorative outcomes than visual factors (Francomano et al., 2022), and their inclusion may introduce additional sources of variability that are difficult to control, such as differences in ambient noise or individual sound sensitivity, potentially confounding the effects of personality and spatial familiarity. To maintain experimental control and ensure that differences in familiarity are attributable solely to landscape features, the study relies exclusively on visual stimuli. Panoramic VR videos were used to simulate realistic visual depth, authentic lighting, and dynamic scene changes, providing an immersive three-dimensional experience validated for assessing landscape preferences and affective responses.

According to previous research (Bazrafshan et al., 2023; Browning et al., 2020), our research team used the Insta360 3X camera to capture 2-min panoramic videos in both Area A and B at 5.7K high resolution at the end of June 2024. The videos were played via high-fidelity VR headsets (HTC VIVE Pro Eye) using Tobii Pro Lab software. Prior to the experiment, two researchers with expertise in visual design independently tested the system to ensure distortion-free playback and sufficient visual clarity to support immersive experiences. The camera was set at 1.2 m to mimic a seated viewer’s perspective, which is common in urban parks. This height matched the viewing angle of participants, who were also seated during the VR experiment.

### 3.2 Participants

Based on an *a priori* power analysis using GPower, a minimum sample size of 46 participants was required for the two-way mixed design under the expected medium effect size (*f* = 0.25), significance level (α = 0.05), and statistical power (1-β = 0.90). In this study, a total of 78 participants were initially recruited through each university’s online forum, with 72 ultimately included in the analysis after excluding those with incomplete or unqualified physiological data (35 females, 37 males; mean age = 22.53 years, range = 18–30). All participants were students or staff in good physical and mental health, residing in either Area A or Area B (36 per area), and had lived on their respective campuses for over 1 year. This sample was selected to control for potential confounding effects of age, gender, and educational background, which could influence the experimental outcomes, as well as due to the digital proficiency required for VR operation and the streamlined ethical approval process of campus committees. All participants possessed tertiary-level education, with many holding academic diplomas. To control for familiarity, participants from Area A had not visited Area B, and vice versa (Table 1). The study was approved by the ethics committee of the corespondence author’s affiliated institution. Participants were compensated 50 RMB for approximately 50 min of the experiment. Socio-demographic information was collected during the recruitment phase.

**TABLE 1:** Participants’ familiarity with each video.

Participants’ category	High-familiarity scene	Low-familiarity scene
Participants from Area A	Area A	Area B
Participants from Area B	Area B	Area A

To control for order effects, we used a counterbalanced design in which participants were exposed to both Area A and Area B. Half experienced Area A first and then Area B, while the other half started with Area B and then Area A. This approach allows us to isolate the effect of scene familiarity on affective responses, independent of exposure order or other confounding variables. The design also ensures an even distribution of gender, age, and other relevant variables across conditions, enabling a fair comparison between high and low-familiarity scenes and minimizing experimental bias.

Participants were recruited through campus posters and online advertisements. All had normal or corrected-to-normal and color vision and had not previously participated in similar experiments. Before the experiment, participants received a comprehensive briefing on the study’s purpose, procedures, and potential risks. They were informed of their right to withdraw, ensuring their autonomy and adherence to ethical standards.

### 3.3 Variables

#### 3.3.1 Explanatory variables

The independent variables in this study include familiarity with urban parks and the big five traits, which are used to elucidate and forecast variations in the dependent variables.

**FIGURE 2:**
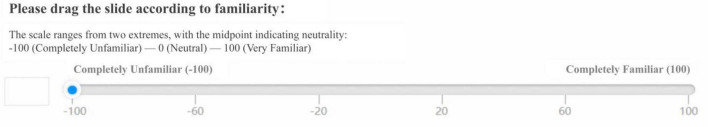
The diagram of the familiarity slider (Illustrated in English; Chinese version applied during actual experimental procedures).

Familiarity was gauged by having participants select a score on a slider ranging from −100 to 100 (Figure 2). By definition, for participants from Area A, the Area A scene is considered more familiar, whereas the Area B scene is less so. The opposite is true for participants from Area B.

The brief version of the Chinese Big Five Personality Inventory (CBF-PI-B) (Meng-Cheng et al., 2011), which comprises 40 items, has demonstrated strong psychometric properties, including reliability and validity, and outperforms similar tools globally. This scale yields scores across five personality dimensions: neuroticism, conscientiousness, agreeableness, openness, and extraversion.

#### 3.3.2 Dependent variables

Typically, affective responses are quantified using subjective self-report questionnaires and objective physiological measurements. As a subjective self-report questionnaire, the Positive and Negative Affect Schedule (PANAS) has been consistently recognized as a reliable measure of subjective emotional states in psychology across diverse contexts (Roemer and Medvedev, 2022). Its availability in multiple languages and its use in cross-cultural studies have solidified its status as a prevalent evaluation tool (Carvalho et al., 2013). The PANAS scale, consisting of 20 items rated on a 5-point Likert scale, assesses participants’ PA and NA following visual stimuli. Specifically, the PA subscale evaluates high energy and concentration through adjectives such as “Interested,” “Excited,” “Strong,” “Enthusiastic,” “Proud,” “Alert,” “Inspired,” “Determined,” “Attentive,” and “Active.” Elevated PA scores reflect high energy and wellbeing, while lower scores are associated with affective detachment and depressive episodes (Lopez and Snyder, 2004). Conversely, the NA subscale quantifies subjective distress and unpleasurable engagement using items including “Distressed,” “Upset,” “Guilty,” “Scared,” “Hostile,” “Irritable,” “Ashamed,” “Nervous,” “Jittery,” and “Afraid.” High NA scores indicate distress and negative emotions, often related to anxiety (Watson et al., 1988), while lower scores denote a calm state. Therefore, low PA and high NA are regarded as markers of poor mental health.

Objective physiological measurements serve as a direct and quantifiable means of assessing the body’s physiological responses to diverse stimuli, with heart rate being a key indicator due to its direct correlation with cardiovascular function and stress levels. Heart rate variability (HRV), our study’s primary biomarker, illuminates psychological states like stress due to its link with the autonomic nervous system. HRV captures the balance between sympathetic and parasympathetic activity, indicating adaptability. Natural environments can reduce stress and heart rate by stimulating the parasympathetic system and lowering cortisol, making HRV a valuable metric for relaxation and stress reduction (Duan et al., 2024; Liu L. et al., 2022; Sun et al., 2023; Zhang, 2023).

Photoplethysmography (PPG) offers a non-invasive, continuous, and accessible means of monitoring HRV, which is crucial for assessing the dynamic responses of the autonomic nervous system to various physiological and psychological challenges (Benchekroun et al., 2022; Pinheiro et al., 2016). PPG data is derived from the pulse interval time series in human pulse signals and is processed to extract HRV, which captures the minute fluctuations in instantaneous heart rate between successive beats. Standard analysis methods for PPG data include time domain and frequency domain analyses, which can extract features related to various physiological mechanisms. In this study, we focused on three-time domain metrics from HRV analysis—Average Heart Rate (AVHR), Standard Deviation of the N-N Interval (SDNN), and Root Mean Square of Successive Differences (RMSSD)—for our data analysis (Table 2). These indices are directly obtainable through the PsychTech platform, which is in real-time sync with the PsychTech wristband.

**TABLE 2:** Main HRV metrics in this study.

Metrics		Characterization
AVHR	Average heart rate	AVHR increases, indicating increased heart rate, affective arousal, and stress
SDNN	Standard deviation of NN intervals	Reflecting the sympathetic nerve activation information, SDNN increased, indicating that the individual’s ability to adapt to the environment increased
RMSSD	Root mean square of successive differences	Reflecting parasympathetic nerve activation information, RMSSD increased, indicating a decrease in stress level, cognitive load, and an increase in positive emotions

In summary, integrating subjective self-report questionnaires and objective physiological measurements offers a comprehensive understanding of affective responses. These methods not only elucidate the complexity of affective states but also provide a scientific foundation for affective regulation and stress management strategies.

### 3.4 Devices

**FIGURE 3:**
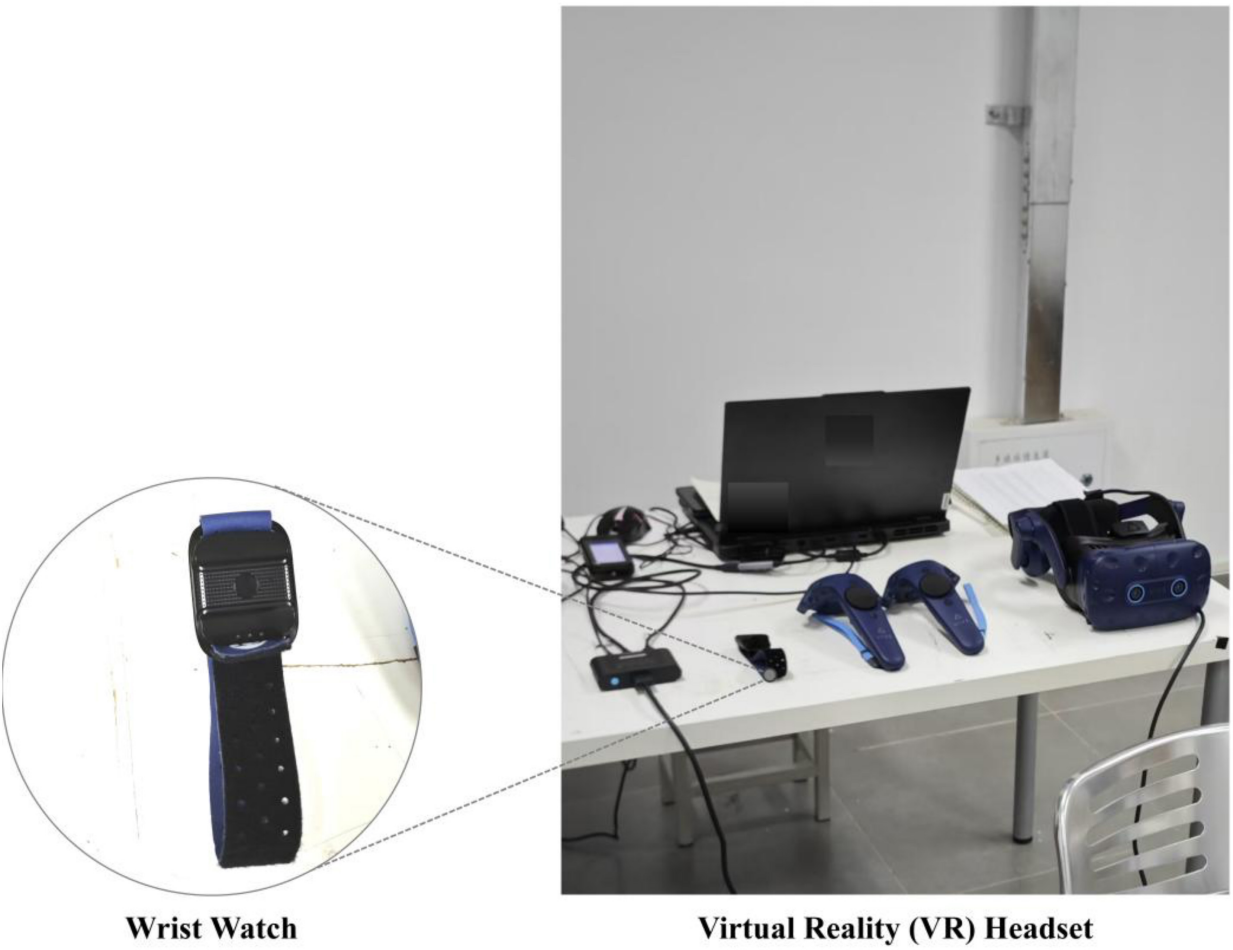
Experimental equipment.

The experiment utilized the HTC VIVE Pro Eye head-mounted display, cables, and a high-performance laptop (Figure 3). A portable wristwatch by PsychTech was employed to measure HRV through PPG signals. PPG detects HRV by quantifying microvascular blood flow oscillations influenced by the autonomic nervous system. This method employs an LED to emit light onto the skin, with the transmitted light being partially absorbed by hemoglobin in the blood. The cardiac cycle-induced changes in blood volume result in detectable variations in light absorption, which are captured by a photodiode. The wristwatch was securely attached to the non-dominant wrist to reduce motion artifacts. The PPG sensor on the wristwatch functioned at a 20 Hz sampling rate, employing a 532 nm wavelength of reflected green light for accurate measurements.

### 3.5 Procedure

**FIGURE 4:**
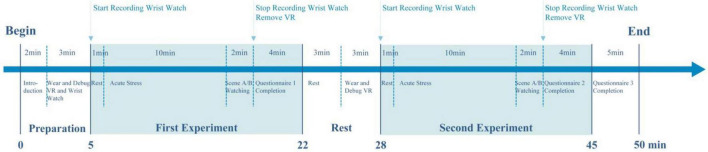
Experimental procedure.

The experimental procedure is depicted in Figure 4. The study consisted of two sessions, each adhering to a uniform protocol. A 3-min rest was included between sessions to mitigate potential visual fatigue associated with VR. This duration is consistent with standard psychophysiological protocols for acute stress tasks, allowing participants’ autonomic nervous system activity, such as heart rate and blood pressure, return to baseline levels before the presentation of the next stimulus (Linden et al., 1997). Each session commenced with a 1-min rest period for participants to adjust to the VR environment. Participants then underwent an acute stressor, following the Trier Social Stress Test—Modified (TSST-M) protocol (Yim et al., 2010), which involved a public speaking task on a specific topic and a mental arithmetic task with problems that differed between sessions. Afterward, participants viewed a 2-min panoramic video. This duration was selected based on empirical studies indicating that 1–5 min of nature exposure is sufficient to elicit immediate affective responses (Browning et al., 2020; McMahan and Estes, 2015). Physiological protocols confirm that visual stimulation as brief as 90 s triggers significant autonomic changes (Song et al., 2018), and ultra-short-term HRV recordings have been validated for data accuracy (Munoz et al., 2015). Furthermore, limiting exposure to 2 min helps minimize the risk of VR-induced cybersickness, ensuring that participants’ reported affect is due to the environment rather than physical discomfort.

Post-video, participants removed their VR headsets and immediately completed the PANAS, assessing the familiarity of the scene they had experienced. Demographic information and personality trait test results were collected at the end of the experiment to enhance the analysis and interpretation of the findings.

### 3.6 Data analysis

The study began with a quality assessment of both independent and dependent variables. Statistical tests were then selected based on the normality of the data distribution (Table 3). For normally distributed data, the *t*-test is a standard method to determine if there is a significant difference in means between two groups. The independent samples *t*-test compares two unrelated groups, while the paired samples *t*-test is used for related groups. Analysis of Variance (ANOVA) is preferred for comparing means across three or more groups. Pearson’s correlation coefficient was used to examine the linear relationship between two variables.

**TABLE 3:** Various statistical test methods.

Statistical test methods	Normal distribution	Not normal distribution
Two independent sample groups	Independent-Sample *T* Test	Mann–Whitney *U* Test
Two related sample groups	Paired *T*-test	Wilcoxon Signed-Rank Test
≥ 3 Independent sample groups	Analysis of variance, ANOVA	Kruskal–Wallis *H* Test
Correlation between two variables	Pearson correlation coefficient	Spearman rank correlation

Non-parametric tests were employed for data that did not meet normality assumptions or had significant variance inequality. The Mann-Whitney U Test is an alternative to the independent samples *t*-test, and the Wilcoxon Signed-Rank Test is used instead of the paired samples *t*-test. The Kruskal–Wallis *H* Test is the non-parametric equivalent to ANOVA for comparing medians among multiple groups. Spearman’s rank correlation coefficient was used to correlate two non-parametric variables.

Furthermore, multiple linear regression analysis is commonly used to investigate linear relationships between multiple independent variables and a single dependent variable. This statistical technique predicts or explains the relationship between a continuous dependent variable and two or more independent variables. It assesses the individual impact of each independent variable on the dependent variable and accounts for interactions between the independent variables. Several key parameters must be considered to evaluate the model’s performance (Table 4). A well-performing model typically exhibits high fit, statistical significance, and residual characteristics that align with assumptions (residuals should be normally distributed without autocorrelation or multicollinearity). Such a model accurately predicts the dependent variable and provides profound insights into the research question.

**TABLE 4:** Parameters of multiple linear regression model.

Category	Parameter	Details
Explain the degree of fitting	*R* ^2^	Explain the proportion of variance in the dependent variable explained by the model’s independent variables. The closer the *R*^2^ value is to 1, the better the model fits.
	Adj. *R*^2^	Consider the number of independent variables
Explain the significance	*F*-statistic	Test the significance of the model, i.e., whether all independent variables collectively impact the dependent variable. A larger value indicates a more significant model overall.
	Prob (*F*-statistic)	When *p* < 0.05, the model is generally considered statistically significant.
Explain the residual characteristics	Omnibus	Test whether the model residuals follow a normal distribution.
	Prob (Omnibus)	When Prob (Omnibus) > 0.05, the residuals can be considered to be normally distributed.
	Durbin-Watson	When the Durbin-Watson statistic is close to 2, there is no autocorrelation among the residuals.
	Cond. No.	When the Condition Number (Cond. No.) is < 100, multicollinearity is generally not a problem.

## 4 Results

### 4.1 Quality assessment of the experiment

Given the non-normal distribution of the data, we utilized the Mann–Whitney *U* Test to assess participants’ familiarity ratings of each scene. The results revealed a significant difference in familiarity between the two scenes (*p* < 0.01), with the high-familiarity scene averaging 87.50 and the low-familiarity scene averaging −57.56, confirming the substantial variation in familiarity among participants, thereby validating the experimental design.

Additionally, Table 5 demonstrates a correlation between the PANAS scale and HRV metrics. The data indicate that the AVHR, SDNN, and RMSSD significantly correlate with NA but not PA in the PANAS scale. Specifically, AVHR correlated positively with NA, while SDNN and RMSSD correlated negatively. This further substantiates the relationship between physiological indicators and NA, reinforcing the experiment’s effectiveness.

**TABLE 5:** Correlation between the PANAS scale and HRV metrics.

Metrics		PA Correlation coefficient (*P*-value)	NA Correlation coefficient (*P*-value)
HRV metrics	AVHR	0.085 (*p* = 0.313)	**0.188 (*p* = 0.024)**
	SDNN	−0.028(*p* = 0.741)	**−0.200 (*p* = 0.016)**
	RMSSD	0.006 (*p* = 0.940)	**−0.171 (*p* = 0.040)**

Bold values indicate statistically significant results (*p* < 0.05).

### 4.2 The influence of personality traits on affective responses

This section examines the relationship between the Big Five personality traits and participants’ responses, integrating both subjective psychological states (PANAS scale) and objective physiological indicators (HRV metrics). Analyzing these links is essential for understanding the mechanisms of person-environment interaction in urban parks.

#### 4.2.1 Correlation analysis of personality traits and PANAS scores

Spearman’s rank correlation analyses were conducted to examine the associations between the Big Five personality traits and affective responses measured by the Positive and Negative Affect Schedule (PANAS). Correlations were first examined at the level of overall positive affect (PA) and negative affect (NA), followed by item-level analyses (Table 6).

**TABLE 6:** Correlation between big five personality traits and PANAS scores.

Personality traits	PA Correlation coefficient (*P*-value)		NA Correlation coefficient (*P*-value)	
	Total PA	Significant PA Items	Total NA	Significant NA Items
Neuroticism	0.096 (*p* = 0.420)	**Alert: 0.393 (*p* = 0.001)**	**0.385 (*p* = 0.001)**	**Ashamed: 0.274 (*p* = 0.020)** **Nervous: 0.470 (*p* < 0.001)** **Jittery: 0.467 (*p* < 0.001)** **Afraid: 0.383 (*p* < 0.001)**
Conscientiousness	−0.041 (*p* = 0.731)		−0.231 (*p* = 0.051)	**Guilty:−0.359 (*p* = 0.002)** **Afraid:−0.243 (*p* = 0.040)**
Agreeableness	0.073 (*p* = 0.544)	**Excited: 0.265 (*p* = 0.025)** **Alert: −0.233 (*p* = 0.049)**	−0.210 (*p* = 0.077)	
Openness	0.149 (*p* = 0.212)	**Interested: 0.244 (*p* = 0.039)** **Proud: 0.252 (*p* = 0.033)**	**−0.300 (*p* = 0.011)**	**Distressed:−0.239 (*p* = 0.044)** **Nervous:−0.244 (*p* = 0.039)** **Jittery: −0.309 (*p* = 0.008)** **Afraid:−0.269 (*p* = 0.023)**
Extraversion	**0.294 (*p* = 0.012)**	**Interested: 0.236 (*p* = 0.046)** **Excited: 0.314 (*p* = 0.007)** **Strong: 0.245 (*p* = 0.038)** **Enthusiastic: 0.353 (*p* = 0.002)** **Proud: 0.384 (*p* = 0.001)** **Inspired: 0.251 (*p* = 0.033)** **Active: 0.281 (*p* = 0.017)**	−0.053 (*p* = 0.659)	

Bold values indicate statistically significant results (*p* < 0.05).

(1) Overall Positive and Negative Affect: At the aggregate level, extraversion was significantly positively correlated with overall PA (*r* = 0.294, *p* = 0.012), indicating that individuals with higher levels of extraversion tended to report higher positive affect. The other four personality traits (neuroticism, conscientiousness, agreeableness, and openness) were not significantly associated with overall PA (*p*s > 0.05). In terms of overall NA, neuroticism was significantly positively correlated with NA (*r* = 0.385, *p* = 0.001), suggesting that individuals with higher levels of neuroticism experienced higher negative affect. Openness was significantly negatively correlated with NA (*r* = −0.300, *p* = 0.011), indicating that individuals with higher openness experienced lower negative affect. Conscientiousness showed a marginally significant negative correlation with NA (*r* = −0.231, *p* = 0.051), while extraversion and agreeableness were not significantly related to overall NA (*p* > 0.05).
(2) Item-Level Affect: At the item level, neuroticism was positively associated with several negative affect items, including alert (*r* = 0.393, *p* = 0.001), ashamed (*r* = 0.274, *p* = 0.020), nervous (*r* = 0.470, *p* < 0.001), jittery (*r* = 0.467, *p* < 0.001), and afraid (*r* = 0.383, *p* < 0.001). Conscientiousness was negatively correlated with guilty (*r* = −0.359, *p* = 0.002) and afraid (*r* = −0.243, *p* = 0.040), indicating that more conscientious individuals tended to experience lower levels of these negative emotions. Agreeableness was positively associated with excited (*r* = 0.265, *p* = 0.025) and negatively associated with alert (*r* = −0.233, *p* = 0.049). Openness was positively correlated with interested (*r* = 0.244, *p* = 0.039) and proud (*r* = 0.252, *p* = 0.033), and negatively correlated with distressed (*r* = −0.239, *p* = 0.044), nervous (*r* = −0.244, *p* = 0.039), jittery (*r* = −0.309, *p* = 0.008), and afraid (*r* = −0.269, *p* = 0.023). Extraversion was positively correlated with several positive affect items, including interested (*r* = 0.236, *p* = 0.046), excited (*r* = 0.314, *p* = 0.007), strong (*r* = 0.245, *p* = 0.038), enthusiastic (*r* = 0.353, *p* = 0.002), proud (*r* = 0.384, *p* = 0.001), inspired (*r* = 0.251, *p* = 0.033), and active (*r* = 0.281, *p* = 0.017).

These findings indicate that extraversion and openness were generally associated with higher positive affect, whereas neuroticism was primarily related to elevated negative affect. Conscientiousness and agreeableness showed more selective associations with specific negative and positive affect items.

#### 4.2.2 Correlation analysis of personality traits and HRV metrics

To examine the potential influence of personality traits on physiological responses, correlation analyses were conducted between the Big Five personality scores and HRV metrics (AVHR, SDNN, and RMSSD). The results revealed no significant correlations between any of the personality traits and the HRV indicators. These findings suggest that while personality traits significantly moderate cognitive and affective appraisals of the environment, the physiological responses to urban parks, as measured by HRV, appear to be relatively universal and consistent across different personality types.

### 4.3 The influence of urban parks’ familiarity on affective responses

This study sought to examine how familiarity with urban parks influences affective responses. We used the PANAS scale to quantify affective differences between the two scenes. We then delved deeper into the effects of scene familiarity by analyzing physiological data.

#### 4.3.1 Analysis of affective responses using the PANAS scale

To investigate the influence of scene familiarity on the PANAS results, we first quantitatively analyzed affective differences across scenes with varying familiarity. Since NA and their sub-item scores were non-normally distributed, we used the Wilcoxon Signed-Rank Test for non-parametric analysis on all data except for PA, which was assessed with a paired samples *t*-test. This approach aimed to identify significant differences among the 20 items measured by the PANAS scale. The results, as presented in Table 7, indicate the following significant differences in the PANAS results between two scenes:

**TABLE 7:** Data analysis results of PANAS scale across familiarity levels.

PANAS item		High-familiarity scene				Low-familiarity scene				Difference	
		Mean		Std		Mean		Std		*P*-value	
		Total	Item	Total	Item	Total	Item	Total	Item	Total	Item
PA	Interested	24.54	3.01	6.33	0.94	25.42	3.19	6.4	1.00	0.18	0.147
	Excited		2.40		0.93		2.53		0.95		0.233
	Strong		2.14		0.98		2.10		0.91		0.614
	Enthusiastic		2.58		0.98		2.68		0.90		0.384
	Proud		2.36		1.03		2.10		0.95		**0.043**
	Alert		1.65		0.87		2.13		1.07		**0.001**
	Inspired		2.21		1.02		2.24		1.11		0.770
	Determined		2.22		0.97		2.36		1.03		0.217
	Attentive		3.14		0.95		3.11		0.97		0.775
	Active		2.82		0.94		2.99		0.94		0.170
NA	Distressed	13.61	1.57	5.33	0.93	14.46	1.79	4.85	0.92	**0.03**	0.068
	Upset		1.50		0.82		1.50		0.75		0.978
	Guilty		1.26		0.63		1.25		0.55		0.806
	Scared		1.18		0.54		1.19		0.46		0.885
	Hostile		1.15		0.52		1.14		0.35		0.725
	Irritable		1.18		0.48		1.22		0.51		0.285
	Ashamed		1.26		0.69		1.40		0.71		0.054
	Nervous		1.67		0.96		1.94		0.87		**0.006**
	Jittery		1.60		0.88		1.71		0.98		0.202
	Afraid		1.24		0.54		1.31		0.57		0.335

Bold values indicate statistically significant results (*p* < 0.05).

(1) There was a statistically significant increase in NA in the low-familiarity scene compared to the high-familiarity scene (*p* < 0.05), with a 6.25% rise, suggesting that participants experienced more distress in low-familiarity environments.
(2) “Alert” was 29.09% higher in the low-familiarity scene than in the high-familiarity scene (*p* < 0.05).
(3) “Nervous” increased by 16.17% in the low-familiarity scene compared to the high-familiarity scene (*p* < 0.05).

#### 4.3.2 Analysis of physiological responses using HRV metrics

Given the non-normal distribution of main HRV metrics (AVHR, SDNN, and RMSSD), we applied the Wilcoxon Signed-Rank Test to assess the statistical significance of differences between the different familiarity scenes (Table 8). The results indicated significant differences between stressors and prepared scenes (*p* < 0.001), suggesting that parks with varying levels of familiarity can effectively reduce affective arousal and stress, enhancing the autonomic nervous system’s adaptability. Compared with the high-familiarity scene, the HRV metrics for the low-familiarity scene exhibited a higher mean AVHR, as well as lower mean values for SDNN and RMSSD. However, no statistically significant differences were observed between the high-familiarity and low-familiarity scenes.

**TABLE 8:** Comparative analysis of PPG data under different familiarity levels.

HRV metrics	Stress	High-familiarity scene	Low-familiarity scene	Stress vs. high-familiarity scene	Stress vs. low-familiarity scene	High vs. low-familiarity scene
AVHR	95.80 ± 13.48	75.12 ± 15.47	76.60 ± 15.47	***p* < 0.01**	***p* < 0.01**	*p* = 0.476
SDNN	21.94 ± 7.91	72.80 ± 37.11	65.64 ± 24.89	***p* < 0.01**	***p* < 0.01**	*p* = 0.063
RMSSD	22.39 ± 8.94	75.40 ± 46.84	65.34 ± 30.00	***p* < 0.01**	***p* < 0.01**	*p* = 0.052

Bold values indicate statistically significant results (*p* < 0.05).

### 4.4 The role of urban parks’ familiarity in personality traits’ effects on affective responses

This study investigates how environmental familiarity acts as a critical moderator that shapes the relationship between the Big Five personality traits and affective responses. While personality traits act as predictors of emotion, the analysis reveals that familiarity determines the activation and intensity of these influences.

To systematically elucidate this moderating role, the analysis follows a three-step approach. Initially, a correlation analysis was performed between the Big Five scores and participants’ PANAS results and three types of HRV data across both scenes to identify how familiarity levels modulate the influence of specific personality traits. It performs a correlation analysis between the scores of the five personality traits and participants’ PANAS results in two scenes and three types of HRV data. This analysis elucidates how familiarity shapes affective responses to different personality traits. Subsequently, the study compares affective response differences among groups with high and low scores on various personality traits to identify which subgroups are most sensitive to changes in environmental familiarity. Lastly, a multiple linear regression model is used to evaluate the predictive power of personality traits as independent variables on NA in low-familiarity urban park environments.

#### 4.4.1 Analysis of personality traits and PANAS scale across familiarity degrees

##### 4.4.1.1 Correlation analysis between personality traits and the PANAS across familiarity degrees

In this study, we conducted the Spearman’s rank correlation analysis between the scores of the big five traits and the PANAS results in scenes of varying familiarity, with results presented in Table 9:

**TABLE 9:** Correlation between personality traits and the PANAS across familiarity degrees.

Personality traits	Familiarity	PA Correlation coefficient (*P*-value)		NA Correlation coefficient (*P*-value)	
		Total PA	Significant PA Items	Total NA	Significant NA Items
Neuroticism	High	−0.001(*p* = 0.994)	**Alert: 0.416 (*p* < 0.001)**	**0.332 (*p* = 0.004)**	**Guilty: 0.253 (*p* = 0.032)** **Hostile: 0.252 (*p* = 0.033)** **Ashamed: 0.251 (*p* = 0.033)** **Nervous: 0.392 (*p* = 0.001)** **Jittery: 0.415 (*p* < 0.001)** **Afraid: 0.274 (*p* = 0.020)**
	Low	0.116 (*p* = 0.331)	**Alert: 0.235 (*p* = 0.047)** **Attentive: 0.252 (*p* = 0.032)**	**0.394 (*p* = 0.001)**	**Ashamed: 0.266 (*p* = 0.024)** **Nervous: 0.461 (*p* < 0.001)** **Jittery: 0.400 (*p* < 0.001)** **Afraid: 0.321 (*p* = 0.006)**
Conscientiousness	High	0.108 (*p* = 0.368)	**Alert: −0.263 (*p* = 0.025)**	−0.185 (*p* = 0.119)	
	Low	−0.136 (*p* = 0.255)		**−0.269 (*p* = 0.022)**	**Guilty: −0.288 (*p* = 0.014)** **Ashamed: 0.282 (*p* = 0.016)**
Agreeableness	High	0.198 (*p* = 0.096)	**Excited: 0.318 (*p* = 0.006)** **Inspired: 0.237 (*p* = 0.045)**	−0.144 (*p* = 0.227)	
	Low	−0.010 (*p* = 0.930)		−0.184 (*p* = 0.122)	
Openness	High	0.203 (*p* = 0.087)	**Excited: 0.273 (*p* = 0.020)**	−0.116 (*p* = 0.330)	**Jittery: −0.238 (*p* = 0.044)**
	Low	0.115 (*p* = 0.338)		**−0.365 (*p* = 0.002)**	**Upset: −0.255 (*p* = 0.031)** **Nervous: −0.259 (*p* = 0.028)** **Jittery: −0.276 (*p* = 0.019)** **Afraid: −0.334 (*p* = 0.004)** **Distressed: 0.291 (*p* = 0.013)**
Extraversion	High	**0.254 (*p* = 0.032)**	**Excited: 0.276 (*p* = 0.019)** **Enthusiastic: 0.306 (*p* = 0.009)** **Proud: 0.308 (*p* = 0.008)** **Active: 0.269 (*p* = 0.022)**	−0.035 (*p* = 0.767)	
	Low	**0.307 (*p* = 0.009)**	**Interested: 0.249 (*p* = 0.035)** **Strong: 0.328 (*p* = 0.005)** **Enthusiastic: 0.257 (*p* = 0.030)** **Proud: 0.324 (*p* = 0.005)**	−0.072 (*p* = 0.548)	

Bold values indicate statistically significant results (*p* < 0.05).

1) Neuroticism was positively correlated with total negative affect in both high-familiarity (*r* = 0.332, *p* = 0.004) and low-familiarity scenes (*r* = 0.394, *p* = 0.001). At the item level, neuroticism showed significant positive correlations with “Nervous,” “Jittery,” and “Afraid” in both contexts, with a stronger association for “Nervous” in the low-familiarity condition (*r* = 0.461, *p* < 0.001). “Alert” was positively correlated with neuroticism in both scenes. In the high-familiarity condition, additional significant positive correlations were observed with “Guilty,” “Hostile,” and “Ashamed.” In the low-familiarity condition, neuroticism was also positively correlated with “Ashamed.”
2) Conscientiousness was not significantly correlated with total positive affect in either condition. In the high-familiarity scene, it was negatively correlated with “Alert” (*r* = −0.263, *p* = 0.025). In the low-familiarity scene, conscientiousness was negatively associated with total negative affect (*r* = −0.269, *p* = 0.022), as well as with “Guilty” and “Ashamed.”
3) Agreeableness showed a marginal positive correlation with total positive affect in the high-familiarity condition (*r* = 0.198, *p* = 0.096). Significant positive associations with “Excited” and “Inspired” were observed only in the high-familiarity scene. No significant correlations with total negative affect were found under either condition.
4) Openness was negatively correlated with total negative affect in the low-familiarity condition (*r* = −0.365, *p* = 0.002), with significant negative associations with “Upset,” “Nervous,” “Jittery,” “Afraid,” and “Distressed.” In the high-familiarity condition, openness was negatively correlated only with “Jittery.” Additionally, in the low-familiarity scene, openness was positively correlated with “Excited.”
5) Extraversion was positively correlated with total positive affect in both high-familiarity (*r* = 0.254, *p* = 0.032) and low-familiarity scenes (*r* = 0.307, *p* = 0.009). In the high-familiarity condition, significant positive correlations were observed with “Excited,” “Enthusiastic,” “Proud,” and “Active.” In the low-familiarity condition, extraversion was significantly correlated with “Interested,” “Strong,” “Enthusiastic,” and “Proud.” No significant correlations between extraversion and total negative affect were observed.
6) Overall, neuroticism and extraversion demonstrated consistent associations with total negative and positive affect across familiarity levels, whereas openness and conscientiousness showed stronger associations under low familiarity, and agreeableness showed significant associations primarily under high familiarity.

Compare to Table 6, when considering familiarity (Table 9), the correlations between personality traits and PANAS subscales revealed notable modulations. For Neuroticism, high familiarity slightly attenuated the associations with negative affect subscales such as “Guilty,” “Hostile,” and “Ashamed,” while low familiarity strengthened correlations with “Nervous,” “Jittery,” and “Afraid.” Extraversion consistently showed positive correlations with multiple positive affect subscales, including “Excited,” “Enthusiastic,” and “Proud,” regardless of familiarity, with no significant links to negative affect. Conscientiousness and Openness exhibited stronger negative correlations with specific negative affect subscales under low familiarity, particularly “Guilty,” “Ashamed,” “Upset,” “Nervous,” “Jittery,” “Afraid,” and “Distressed.” Agreeableness showed modest positive associations with positive affect subscales such as “Excited’ and “Inspired” only in high familiarity conditions. These results indicate that familiarity modulates the relationship between certain personality traits and specific positive and negative affective responses.

##### 4.4.1.2 PANAS responses of high and low personality groups across familiarity levels

This study investigates how environmental familiarity interacts with extreme personality scores to shape affective responses (Guo et al.; Schueler et al.). By employing a high/low scoring grouping method, we assessed how the contrast between high- and low-familiarity urban parks serves as a differential emotional catalyst for various personality types. We identified the top and bottom 30 individuals for each trait, forming five groups with ten subgroups in total. Wilcoxon Signed-Rank Tests were conducted to examine the differences in affective responses between high- and low-familiarity scenes for each subgroup. The statistical comparisons are detailed in Table 10.

**TABLE 10:** Statistical comparison of PANAS item scores between high- and low-familiarity urban parks across personality groups.

Personality traits	Personality level	Significant difference in PA W (*P*-value), direction	Significant difference in NA W (*P*-value), direction
Neuroticism	High		
	Low	Alert: 0.0 (*p* = 0.001), low-familiarity↑	
Conscientiousness	High	Proud: 26.5 (*p* = 0.008), high-familiarity↑ Alert: 5.5 (*p* = 0.001), low-familiarity↑	
	Low	Enthusiastic: 22.5 (*p* = 0.011), low-familiarity↑ Ashamed: 9.0 (*p* = 0.046), low-familiarity↑ Determined: 14.0 (*p* = 0.003), low-familiarity↑	Nervous: 32.0 (*p* = 0.022), low-familiarity↑
Agreeableness	High	Proud: 46.0 (*p* = 0.023), high-familiarity↑	
	Low	Alert: 44.0 (*p* = 0.034), low-familiarity↑	
Openness	High	Proud: 42.0 (*p* = 0.047), low-Familiarity↑	
	Low	Alert: 24.0 (*p* = 0.006), low-familiarity↑	Ashamed: 0.0 (*p* = 0.015), low-familiarity↑
Extraversion	High		
	Low	Alert: 40.5 (*p* = 0.044), low-familiarity↑	Ashamed: 0.0 (*p* = 0.023), low-familiarity↑ Nervous: 42.5 (*p* = 0.023), low-familiarity↑

↑Indicates the scene with significantly higher scores; W = Wilcoxon test statistic.

1) For Neuroticism, no significant differences were observed in the high-neuroticism group. In the low-neuroticism group, “Alert” scores were significantly higher in the low-familiarity scene (*W* = 0.0, *P* = 0.001), indicating increased alertness under unfamiliar conditions.
2) For Conscientiousness, individuals with high conscientiousness showed significantly higher “Proud” scores in the high-familiarity scene (*W* = 26.5, *p* = 0.008) and higher “Alert” scores in the low-familiarity scene (*W* = 5.5, *p* = 0.001). Among those with low conscientiousness, “Enthusiastic” (*W* = 22.5, *p* = 0.011) and “Determined” (*W* = 14.0, *p* = 0.003) were significantly higher in the low-familiarity scene. Additionally, “Ashamed” (*W* = 9.0, *p* = 0.046) and “Nervous” (*W* = 32.0, *p* = 0.022) were also significantly higher in the low-familiarity condition.
3) For Agreeableness, individuals with high agreeableness reported significantly higher “Proud” scores in the high-familiarity scene (*W* = 46.0, *p* = 0.023). In the low-agreeableness group, “Alert” was significantly higher in the low-familiarity scene (*W* = 44.0, *p* = 0.034).
4) For Openness, participants with high openness exhibited significantly higher “Proud” scores in the low-familiarity scene (*W* = 42.0, *p* = 0.047). Among individuals with low openness, “Alert” (*W* = 24.0, *p* = 0.006) and “Ashamed” (*W* = 0.0, *p* = 0.015) were significantly higher in the low-familiarity condition.
5) For Extraversion, no significant differences were found in the high-extraversion group. In the low-extraversion group, “Alert” (*W* = 40.5, *p* = 0.044), “Ashamed” (*W* = 0.0, *p* = 0.023), and “Nervous” (*W* = 42.5, *p* = 0.023) were significantly higher in the low-familiarity scene.
6) Overall, significant differences between familiarity conditions were more frequently observed in low-level personality groups, particularly for “Alert,” “Ashamed,” and “Nervous,” which tended to be elevated in the low-familiarity scene.

**FIGURE 5:**
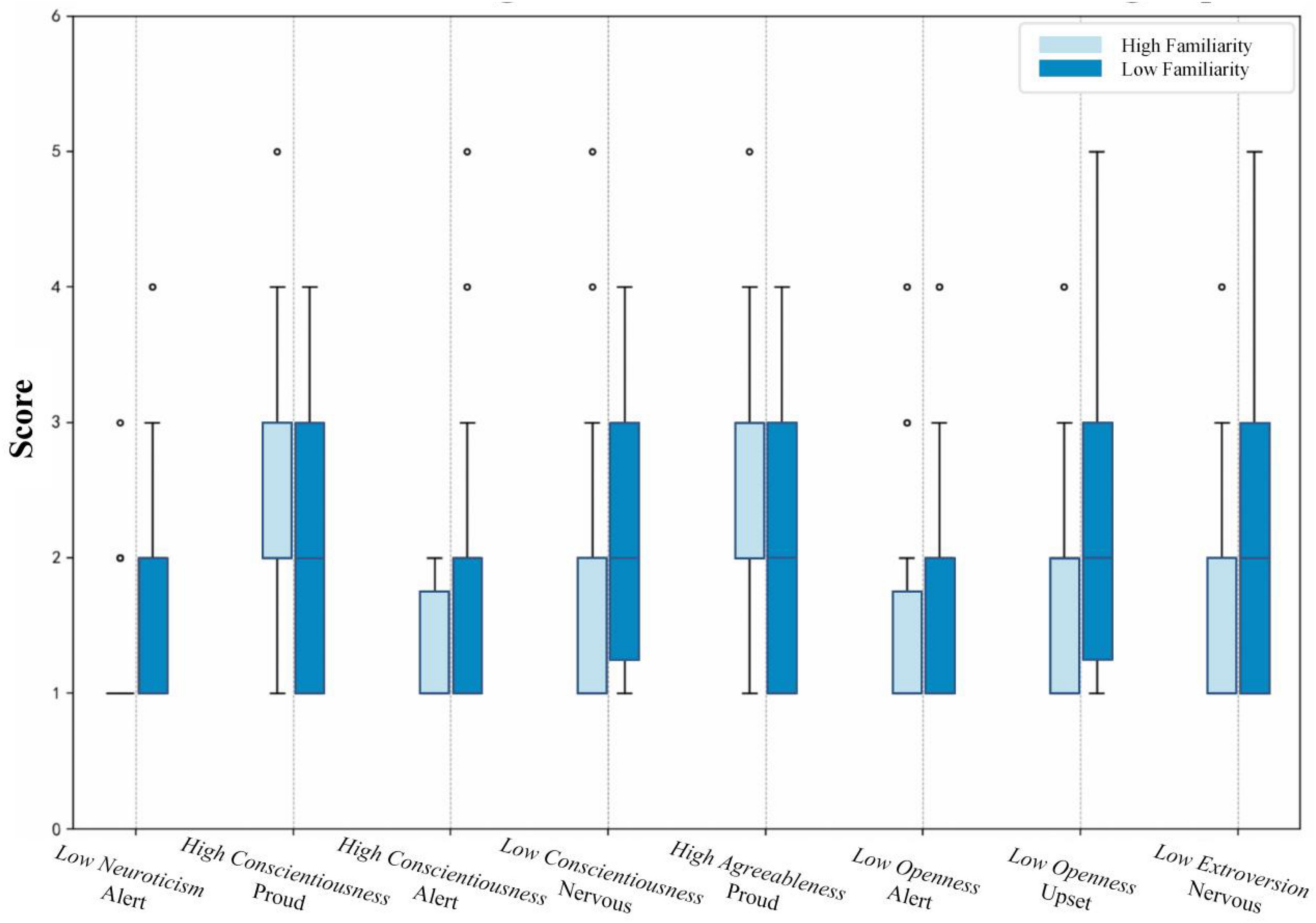
PANAS items with significant differences in different subgroups.

Furthermore, Figure 5 employs box plots to depict the distribution and variation of items that significantly differ across two scenes. Regarding health benefits, the following personality traits promoted an increase in PA items (specifically “Alert”) in the low-familiarity scene compared to the high-familiarity scene:

1) High conscientiousness individuals showed a 55.26% increase.
2) Low neuroticism individuals experienced a 44.74% increase.
3) Low openness individuals exhibited a 26.67% increase.

However, compared to high-familiarity scenes, low openness, low extraversion, and low conscientiousness have exacerbated the increase in NA items:

1) Low openness individuals had a 40.00% increase in “Distressed.”
2) Low extraversion individuals saw a 25.53% increase in “Nervous.”
3) Low conscientiousness individuals experienced a 21.57% increase in “Nervous.”

High conscientiousness and high agreeableness traits were associated with a decrease in PA items (specifically “Proud”):

1) High conscientiousness individuals had a 22.78% decrease.
2) High agreeableness individuals showed a 21.79% decrease.

##### 4.4.1.3 Multiple linear regression: Interaction effects in predicting NA in low-familiarity urban parks

The critical moderating role of familiarity is quantified in the predictive model for low-familiarity settings. Results in 4.2.1 showed a 6.25% increase in NA in the low-familiarity scene compared to the high-familiarity one, prompting us to focus on NA in low-familiarity urban parks. Due to the non-normal distribution of NA data, the Box-Cox transformation was employed to normalize the distribution of the data. We utilized a stepwise regression approach with backward elimination as part of the multiple linear regression analysis to streamline the model by removing predictors that did not significantly contribute to the prediction of the dependent variable, thereby improving the model’s efficiency and reducing the likelihood of overfitting. The final model is:

NA_*low–familiarity urban parks*_ = 0.6220 + 0.0014 × Neuroticism − 0.0012 × Openness.

Key findings from the model results (Table 11) include:

**TABLE 11:** Regression analysis results.

Dep. variable	*R* ^2^	Adj. *R*^2^	*F*-statistic	Prob (*F*-statistic)	
Negative affect	0.260	0.239	12.15	** < 0.0001**	
**Omnibus**	**Prob (Omnibus)**	**Durbin-Watson**	**Cond. No.**		
3.780	0.151	1.984	1.31		
**Variable**	**Coefficient**	**Std. error**	***t*-value**	***P*-value**	**95% Conf. Interval**
Intercept	0.6220	0.000	1454.456	0.000	0.621–0.623
Neuroticism	0.0014	0.000	3.242	0.002	0.001–0.002
Openness	−0.0012	0.000	−2.719	0.008	−0.002 to 0.000

Bold values indicate statistically significant results (*p* < 0.05).

**FIGURE 6:**
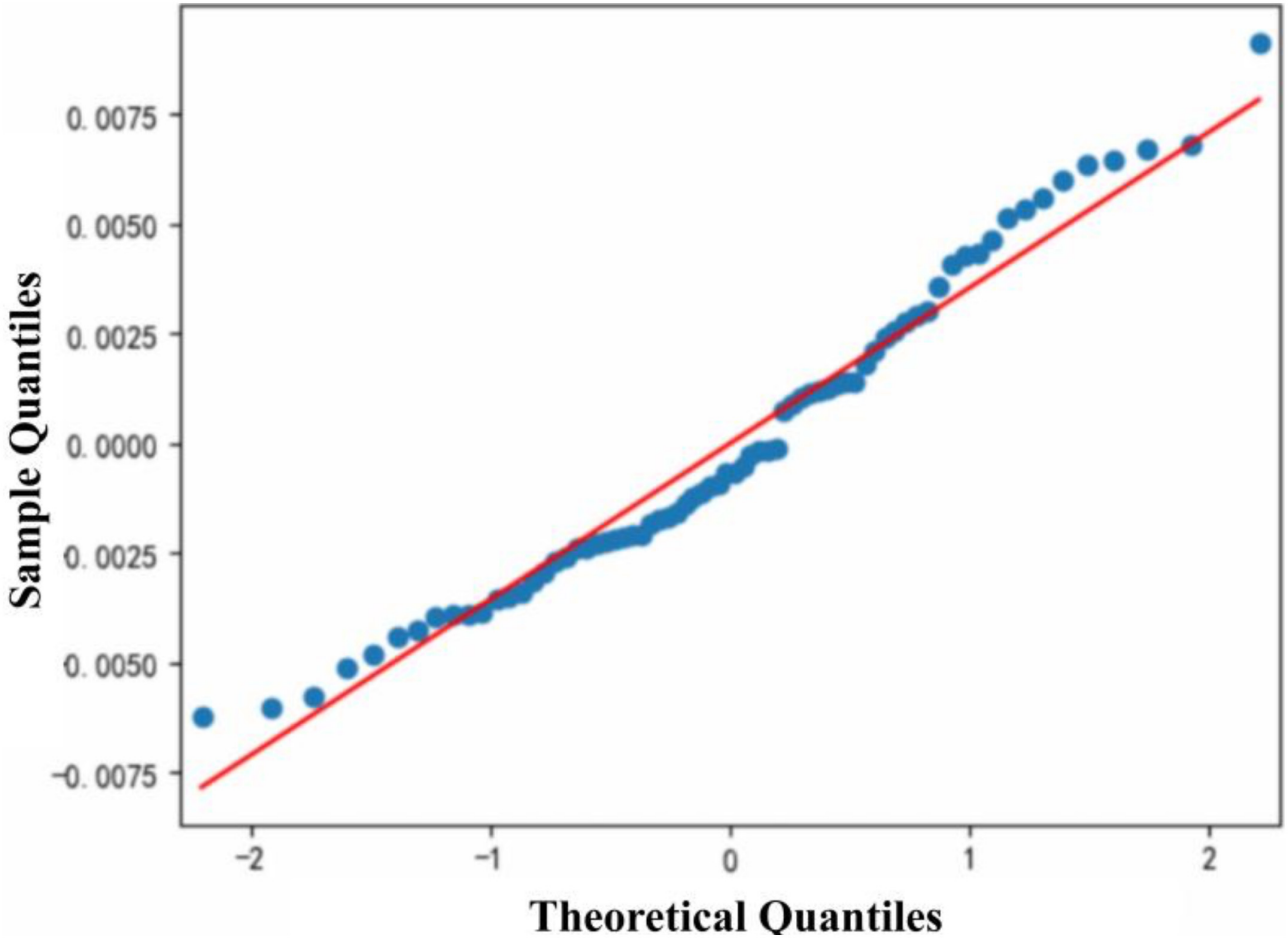
Residual distribution of the model.

1) The model’s *R*^2^ value is 0.260 and is statistically significant (*F* = 12.15, *p* < 0.0001). The residuals of the model meet the basic assumptions of linear regression, including normality of residuals [Prob (Omnibus) > 0.05], absence of autocorrelation (Durbin–Watson value close to 2), and absence of multicollinearity (low condition number). The residual distribution of the model (Figure 6) reveals points closely aligned along the diagonal, suggesting that the residuals are normally distributed. The minimal discrepancy between sample and theoretical quantiles further substantiates the normal distribution of the residuals.
2) Individuals with high neuroticism tend to experience more NA in low-familiarity scenes (β = 0.1056, *p* < 0.01), while those with high openness scores may experience fewer NA (β = −0.0370, *p* < 0.01). These insights enhance our understanding of how personality traits affect affective experiences in specific environments.

#### 4.4.2 Analysis of personality traits and HRV metrics across familiarity degrees

In this study, we initially performed a correlation analysis between the scores of Big Five traits and three HRV metrics (AVHR, SDNN, and RMSSD) in high and low familiarity scenes. The results showed no significant correlations among these variables. To further investigate the relationship between personality traits and PPG data, we identified the top and bottom 30 individuals for each trait, creating five groups with ten subgroups. Comparative analyses of HRV data across these groups in two scenes still revealed no significant differences.

## 5 Discussion

This study examined how individual personality traits and spatial familiarity jointly shape affective responses to urban parks using a VR-based experimental paradigm. By integrating subjective affective assessments (PANAS) with physiological indicators (HRV), the findings indicate that affective restoration in urban parks is not determined solely by environmental exposure, but is substantially modulated by individual differences. Specifically, personality traits showed robust associations with affective outcomes, while spatial familiarity exerted a selective influence on negative affect. Crucially, familiarity moderated the relationship between personality traits and emotional responses, with neuroticism and openness emerging as significant predictors of negative affect in low-familiarity park environments. Together, these findings provide empirical support for a person-environment interaction mechanism in affective restoration, highlighting the role of familiarity in shaping how dispositional traits translate into emotional experiences within urban green spaces.

### 5.1 Personality traits as affective predispositions in urban park experiences

Consistent with Hypothesis 1, extraversion was positively associated with positive affect, whereas neuroticism was positively associated with negative affect in urban parks. These findings support the conceptualization of personality traits as stable affective predispositions that shape emotional responses through attentional allocation, emotional sensitivity, and cognitive appraisal. Extraverted individuals tend to engage more actively with environmental stimuli and exhibit heightened reward sensitivity, amplifying high-arousal positive emotions such as excitement, interest, pride, and enthusiasm. In contrast, neuroticism increases vigilance and threat sensitivity, intensifying discrete negative emotions including nervousness, jitteriness, alertness, shame, and fear. These patterns align with prior research on trait-affect relationships in daily life (Letzring and Adamcik, 2015; Yang et al., 2020) and extend them to naturalistic, restorative contexts.

Openness, conscientiousness, and agreeableness were associated with selective affective responses, highlighting differentiated mechanisms of cognitive appraisal and emotional regulation. High openness was linked to greater interest and pride and lower distress, nervousness, jitteriness, and fear, reflecting curiosity-driven engagement and flexible appraisal that enhances positive affect while buffering negative emotions. Conscientiousness was negatively related to guilt and fear, consistent with self-regulation and goal-directed planning mitigating specific negative affect. Agreeableness was positively associated with excitement and negatively with alertness, suggesting that prosocial orientation and reduced hypervigilance selectively enhance positive emotional experiences. These item-level patterns clarify how personality shapes the intensity and type of discrete affective responses, extending prior findings on conscientiousness and agreeableness (Sirois and Hirsch, 2015; Sella et al., 2023).

The lack of significant correlations between personality traits and HRV metrics indicates that these affective predispositions operate primarily through subjective cognitive-emotional appraisal rather than physiological arousal. While neuroticism and extraversion have been identified as vulnerability and resilience factors in stress regulation (Wardenaar et al., 2014), our findings suggest that in restorative natural contexts, personality mainly shapes subjective affective evaluation rather than autonomic arousal. Taken together, the results demonstrate that personality traits reliably predict individual differences in emotional responses to urban parks, highlighting the theoretical significance of trait-affect relationships in restorative contexts. By linking stable dispositional characteristics to both overall and discrete affective patterns, this study advances understanding of the mechanisms underlying person-environment interactions and reinforces the role of personality as a key determinant of affective experience in naturalistic settings.

### 5.2 Spatial familiarity as an affective regulation mechanism

Supporting Hypothesis 2, spatial familiarity emerged as a significant factor shaping affective responses in urban parks. Participants reported higher negative affect and exhibited trends of increased affective arousal when exposed to low-familiarity park scenes, as evidenced by significant increases in NA, alertness, and nervousness (Table 7). These findings align with cognitive-neuropsychological perspectives suggesting that unfamiliar environments impose greater cognitive demands, elevate uncertainty, and reduce perceived control, which in turn taxes emotional regulation resources (Reber et al., 2004; Harms et al., 2021; Ratcliffe and Korpela, 2016). In contrast, familiar environments provide predictable spatial cues and accumulated experience, enabling efficient cognitive processing and reducing the need for constant environmental monitoring, which facilitates emotional stability and the regulation of negative affect.

Mechanistically, spatial familiarity appears to function as an emotional safety signal. High-familiarity environments are associated with perceived environmental mastery and predictability, which reduce vigilance, support parasympathetic activation, and allow attentional resources to be directed toward positive engagement and reflective cognitive processes (Meyer et al., 2021; Nicolas et al., 2022). Familiar scenes are also more likely to evoke autobiographical memories and stable place meanings, reinforcing emotional comfort and buffering against distress (Lewicka, 2011; Ratcliffe and Korpela, 2016; Bazrafshan et al., 2023). The item-level PANAS results suggest that this buffering effect primarily mitigates specific high-arousal negative states, such as alertness and nervousness, rather than uniformly enhancing positive affect. This distinction clarifies why spatial familiarity influences NA more than PA and underscores its role as a regulatory, rather than generative, mechanism in affective experience.

Physiological data indicated that exposure to urban parks, irrespective of familiarity level, effectively reduced affective arousal relative to stress-inducing conditions, as reflected in HRV improvements across AVHR, SDNN, and RMSSD. However, no significant differences were observed between high- and low-familiarity scenes, suggesting a partial decoupling between subjective affective appraisal and autonomic recovery. This pattern implies that biophilic elements may universally support physiological restoration, whereas subjective emotional responses remain sensitive to cognitive appraisal processes shaped by prior spatial experience. These results integrate with the Person-Place-Process framework (Lewicka, 2011), highlighting familiarity as an integrative mechanism linking individual experience, environmental features, and affective-cognitive regulation.

Overall, these findings advance understanding of the mechanisms through which spatial familiarity modulates emotional responses. By demonstrating that familiarity selectively buffers negative affect and interacts with cognitive appraisal processes, the study provides empirical support for familiarity as an affective regulation mechanism in naturalistic contexts. This emphasizes the theoretical importance of considering accumulated experience and environmental mastery when examining individual differences in affective responses to restorative settings.

### 5.3 Multilevel interaction mechanisms between personality and familiarity

The most theoretically significant contribution of this study lies in elucidating how spatial familiarity moderates personality traits and affective responses, addressing Hypothesis 3. Our findings demonstrate that affective outcomes in urban parks emerge from the dynamic interplay between dispositional traits and the cognitive-emotional demands of the environment, rather than from the additive effects of each factor. This aligns with the Person-Place-Process framework, which conceptualizes familiarity as an integrative mechanism linking personal characteristics, environmental attributes, and affective-cognitive processes (Lewicka, 2011; Kitchin, 1994). Rather than acting independently, personality traits and spatial familiarity jointly shape affective experiences, reflecting a person-environment interaction model consistent with contemporary interactionist perspectives in psychology (Hughes, 2000; Nezlek, 2007).

Mechanistically, spatial familiarity functions as a contextual “switch” that modulates the expression of personality-driven affective tendencies. High familiarity reduces perceptual ambiguity, lowers cognitive load, and enhances predictive processing, thereby enabling individuals to deploy trait-based regulatory strategies effectively. In contrast, low-familiarity environments increase uncertainty, heighten vigilance, and demand greater attentional and emotional regulation effort, which can amplify predisposed negative affect or constrain positive affect (Harms et al., 2021; Ratcliffe and Korpela, 2016; Reber et al., 2004). This mechanism helps explain why participants high in neuroticism showed markedly higher scores on “Alert,” “Nervous,” and “Jittery” in low-familiarity parks, whereas these responses were attenuated in familiar settings. Similarly, individuals with low conscientiousness exhibited elevated “Nervous” and “Ashamed” scores in unfamiliar environments, reflecting the interaction between lower self-regulatory tendencies and environmental novelty.

At the same time, openness and extraversion demonstrated context-dependent facilitation of positive affect. Low-familiarity scenes elicited increased “Alert” and “Excited” scores for certain groups, suggesting that when novelty is cognitively manageable, these traits promote exploratory engagement and heightened positive arousal. Conversely, high conscientiousness and agreeableness were associated with decreased “Proud” scores under low-familiarity conditions, indicating a preference for predictable, controllable settings where mastery can be exercised. These item-level patterns provide concrete evidence for the hierarchical switch mechanism, showing that familiarity can either amplify or buffer trait-specific emotional tendencies depending on the alignment between personality and environmental context.

This multilevel interaction can be conceptualized as a hierarchical regulatory system. At the first level, personality traits establish baseline affective propensities that predispose individuals toward positive or negative emotional patterns (de Raad and Mlaèiæ, 2015; Letzring and Adamcik, 2015). At the second level, spatial familiarity shapes cognitive-emotional interpretation of environmental cues, regulating perceived safety, predictability, and control, which in turn modulate affective responses. The resulting context-sensitive outcomes explain why the same environment may elicit divergent emotional experiences across individuals and why physiological measures, such as HRV, remained largely consistent despite variability in subjective affect, reflecting a partial decoupling of autonomic recovery from appraisal processes (Jeon et al., 2021).

Finally, these insights extend classical restoration theories such as ART and SRT by emphasizing conditional pathways to restoration. Restoration is not purely a property of environmental features but emerges from the coupling of individual differences, prior experience, and environmental structure (Berg, 2020; Bazrafshan et al., 2023). This multilevel perspective provides a theoretical basis for personalized design in urban green spaces and virtual restorative environments (Hedblom et al., 2019; Juliantino et al., 2023), highlighting that a “one-size-fits-all” approach to nature therapy is insufficient; optimal affective outcomes require aligning environmental characteristics with users’ psychological profiles and experiential history, rather than assuming uniform benefits across populations. This insight advances the theoretical discourse on person-environment interactions, providing a foundation for future research on individualized restoration processes, virtual nature simulations, and interventions targeting stress reduction in urban populations (Juliantino et al., 2023; Su et al., 2025).

### 5.4 Limitation and future directions

Despite the theoretical and practical contributions of the present study, several limitations should be acknowledged. First, the sample primarily included highly educated young participants (university students) with moderate familiarity with local parks, which may limit the generalizability of findings to populations with substantially different environmental exposure or cultural backgrounds. Future research could examine cross-cultural or age-diverse samples to test whether personality-familiarity interactions are robust across demographic contexts.

Second, although the study incorporated both self-reported affective measures and physiological indicators (HRV), the physiological effects of familiarity were subtle and not statistically significant. This suggests that psychological appraisal may be more sensitive to spatial familiarity than autonomic responses, or that additional physiological metrics (e.g., cortisol, skin conductance, or neuroimaging data) could better capture the interplay between personality, familiarity, and stress regulation. Future studies integrating multimodal physiological and neurocognitive measures could clarify the mechanisms linking environmental cognition, affective processing, and autonomic adaptation.

Third, the operationalization of spatial familiarity was based on self-reported recognition and experiential frequency, which captures global familiarity but may overlook fine-grained spatial features such as path configuration, vegetation layout, or visual focal points that could differentially influence affective and cognitive responses. Subsequent research could employ more detailed spatial mapping or virtual reality manipulations to disentangle the contribution of specific environmental layers to affective regulation.

Collectively, these limitations highlight opportunities for future research to refine personalized urban park design and virtual restorative environments, ensuring that interventions are attuned to both dispositional and experiential characteristics of users. By systematically investigating multilevel interactions among personality, familiarity, and environmental features, future work can further elucidate the psychological mechanisms underlying individual differences in emotional restoration and guide evidence-based strategies for optimizing urban green space benefits.

## 6 Conclusion

This study investigated how personality traits and spatial familiarity jointly shape affective responses to urban park environments by integrating self-reported emotions with physiological indicators. The findings demonstrate that emotional experiences in urban green spaces are not uniform but vary systematically according to individual dispositions and experiential context.

Across familiarity conditions, personality traits showed stable associations with affect. Neuroticism was consistently related to higher negative affect (NA), while extraversion was associated with higher positive affect (PA), indicating that personality functions as a dispositional baseline for emotional experience. In contrast, conscientiousness and openness exhibited context-dependent effects, becoming more relevant in low-familiarity environments, where unfamiliarity may heighten emotional sensitivity.

Spatial familiarity independently influenced affective responses, primarily by modulating negative affect. Unfamiliar park environments were associated with elevated NA (such as nervousness and unease) and subjective arousal, whereas familiar settings supported greater emotional stability. Notably, high conscientiousness and openness lessen NA in low-familiarity scenes. A multiple linear regression model using the OLS method confirmed neuroticism and openness as significant predictors of NA in low-familiarity urban parks, offering new insights into affective experiences in specific environments. Physiological indicators suggested overall stress reduction during park exposure regardless of familiarity, pointing to a partial dissociation between autonomic regulation and conscious affective appraisal.

Beyond urban park research, these findings have broader implications for fields that employ environmental or spatial experiences to support emotional regulation and wellbeing, including healthcare environments, virtual and augmented reality applications, workplace design, zoned park areas and digital therapeutic interventions. Incorporating individual differences in personality and prior familiarity may enhance the effectiveness of such environments by aligning emotional demands with users’ psychological predispositions. This person-centered perspective provides a transferable framework for designing restorative and supportive environments across both physical and virtual domains.

## Data Availability

The raw data supporting the conclusions of this article will be made available on request to the corresponding author.
